# Taking a nuanced look at adolescent technology use and negative affect: the protective role of preparedness

**DOI:** 10.3389/fpsyt.2023.1015635

**Published:** 2023-05-15

**Authors:** Sean McFarland, Tse Yen Tan, Kalee De France, Jessica D. Hoffmann

**Affiliations:** Yale Center for Emotional Intelligence, Yale University, New Haven, CT, United States

**Keywords:** adolescents, technology, social media, internet, mental health, negative affect, emotions

## Abstract

Adolescents are online more than any other age group, with the majority of their time on social media. Increases in technology use among adolescents have heightened conversations regarding its effects on their negative affect. There have been mixed findings regarding the relationship between technology use and adolescent negative affect; some studies present a negative association or no association, and some show a positive association. To clarify this relationship, we propose moving away from asking only how much adolescents use technology to asking how and what they use it for. We employed the Multidimensional Healthy Technology Use and Social Media Habits Scale (MTECH) and adapted forms of the Positive and Negative Affect Schedule (PANAS) in a sample of 7,234 middle and high school students to assess the extent to which students feel prepared to use technology safely and successfully and whether this impacts the association between the amount of various types of technology they use and their negative affect. We conducted eight moderated regression analyses that, in some models, revealed preparedness had a protective role in the association between technology use and negative affect. In these models, at all levels of technology use, adolescents with higher levels of preparedness experienced lower levels of negative affect than their peers; however, in some instances, this effect was diminished for those using technology with high frequency. These findings support the notion that the association between technology and negative affect is not best modeled as a direct relationship, and instead that we must consider important moderators of this complex association.

## Introduction

Adolescent technology use, and the link between adolescent affective well-being and technology use, has become subject to increasing interest in recent years. In particular, there are rising concerns that technology use may be linked to negative mental health outcomes including increases in suicidal ideation (1). For example, adolescent cyberbullying has been widely studied as one negative factor of technology use (2). Popular media reports have led to concerns, and even panic, that screen time harms children (3), however studies focused on the association between technology use and adolescent affective experiences have, in reality, yielded mixed results. Conflicting evidence has suggested small, negative associations between technology and affect (1), some positive effects [i.e., on social connectedness and mental health: (4)], to little to no positive or negative effects at all (5).

These mixed findings signal the need for more nuance when examining the technology use and negative affect association. A growing body of literature suggests that one-to-one associations between overall technology use (often indicated by hours of screen time) and negative affect may not be the most meaningful approach to understanding how technology use connects to negative affect or adolescent development [e.g., (6–8)]. Instead, there may be important individual difference factors that moderate how, and how strongly, technology use is associated with negative affect. One way to parse out the nuances of technology use is to examine multiple types of technology use—from engagement with social media to gaming to completing assignments for school. There may also be important individual difference factors embedded in the individuals themselves, and how they use technology that could represent moderating influences on the association between technology use and experiences of negative affect. We propose one factor particularly worthy of investigation: preparedness—how well a student feels their school has taught them to use the internet and other digital tools effectively and safely.

When discussing the preparedness of an adolescent to use technology, we are specifically referring to their school-trained preparedness (synonymous with school-trained digital literacy). This is defined as the extent to which a school has adequately prepared its students to succeed when using technology in multiple ways, including for their academics, on social media, and for future career paths. The World Economic Forum reported in 2020 that two of the top 10 skills for future jobs involve an extensive knowledge of technology, and that the top three jobs growing in demand are technological and data-driven fields (9). There have been calls in recent literature for more interventions in schools to increase students’ attitudes and skills in online spaces (10). Practically, this is ensuring that students have enough knowledge to use different software programs to properly complete their schoolwork, or perhaps teaching students how to properly conduct their own research online in pursuit of reliable information. Hague and Payton (11) detail the many benefits of school-wide preparedness to use technology as well as mention some challenges that schools may face in reaching this goal. There have been several studies showcasing various digital literacy interventions in secondary schools and their positive impacts on students (12, 13). For example, Patmanthara and Hidayat (14) found an increase in students’ creativity and critical thinking skills after implementing a school-wide digital literacy intervention. For schools who are able to achieve a high level of preparedness in their students, it is likely that we will observe less negative affect when using technology.

### The current study

We developed the current study to examine the relationship between technology use and adolescent negative affect while accounting for some of the nuance that exists within the realm of technology use. First, we sought to explore the role of preparedness (how well-prepared a student is by their school to use technology) in the relationship between technology use and negative affect. Given popular belief that technology use is associated with negative affect, and existing literature states this association may be changed with more nuanced measurement, we hypothesized that preparedness would significantly moderate the association between the amount adolescents use technology and how much negative affect they report experiencing. This would follow previous research in highlighting the benefits of preparedness on student outcomes.

Second, we sought to evaluate the role of preparedness in different domains of technology use (e.g., social media, video gaming, creating digital content, using technology for school), again exploring their association with negative affect. This aim was more exploratory, as we hypothesized that preparedness might play a different role in this association depending on the domain of technology use. We chose to address the moderating role of preparedness over other potential moderating factors in order to provide guidance to schools on which domains of technology their preparedness efforts can benefit the most, and to highlight the importance of this topic in adolescent education. Many schools are interested in helping their students succeed in using technology, however it can be difficult to tell if their efforts are working, and if they are, identifying which specific domains of technology use see these benefits is relatively unexplored territory. This addresses the dearth of literature that investigates nuanced conceptualizations of technology use that goes beyond the total amount of screentime to better understand *which* technology activities are associated with negative affect, and *how* preparedness might moderate these relationships.

## Method

### Participants and procedure

This study used data from a larger sample of adolescents designed to evaluate youth empowerment and healthy technology use (15). The sample was restricted to students in grades 6–12 enrolled in middle and high schools within the United States. All students within a participating school were invited to take part in the study. In total, 7,234 students from 34 schools within the United States responded to surveys containing items about technology usage. These schools were from across the United States, with 18 representing the Midwest, 7 from the South, 5 representing the West, and 4 from the Northeast. All schools had signed school agreement forms to have their students participate, and followed district guidelines with regards to parent informed consent or parent-opt out procedures. Students also provided informed assent prior to participation, and were aware that their responses were anonymous and that they could skip any question or opt out at any time. Measures were distributed using Qualtrics survey software, and all measures were taken on the same day.

Participants were excluded from the study if they failed to correctly respond to two attention check items (“Choose ‘2’ for this item”; “Choose ‘1’ for this item”), resulting in 666 participants being excluded from further analyses and a final sample of 6,568 participants. Of this sample,41.8% identified as male, 42.8% identified as female, 2.5% identified as non-binary, and 0.9% of participants chose not to report their gender. The sample was predominately White (64.5%), with 12% identifying as Hispanic/Latinx, 2.8% Biracial/Multiracial, 4.6% Asian/Asian-American, 4.3% Black/African-American, 1.6% Middle Eastern, 4.3% another race/ethnicity, and 2.3% who did not report their race/ethnicity. Participant age ranged from 11 to 21 (mean = 13.27, *SD* = 1.68) Data collection occurred over 15 months from December 2020 to February 2022.

### Materials

#### Multidimensional healthy technology use and social media habits scale

To measure Technology Use in a nuanced lens, we employed the Multidimensional Healthy Technology Use and Social Media Habits Scale (MTECH) (16). The scale begins by obtaining the frequency in which adolescents use 7 different types of technology. Participants were given the following instructions: “There are many ways to engage with technology and the Internet. How often do you do each of the following?” Sample items include “Play video games, computer games, or games on your phone,” and “Look at content on social media (e.g., watch Instagram stories, scroll through feeds).” Participants rated these items from 1 (“*Never*”) to 4 (“*All the time*”). We averaged participants’ scores within each item to produce the average level of Technology Use for each domain, with higher scores representing a higher frequency of that type of Technology Use.

#### Preparedness

Preparedness was assessed using the Preparedness subscale of the MTECH (16, 17). Participants rated 4 items on a scale from 1 (*“Strongly disagree”*) to 4 (*“Strongly agree”*). The 4 items were as follows: (1) “My school provides technology classes (e.g., coding, digital design) that will help me get a job”; (2); “My school has helped me understand how technology can both benefit learning and sometimes hurt”; (3) “I feel prepared by my school for a future in which many jobs will require technology skills”; (4) “My school has taught us how to access the benefits of the internet while avoiding the dangers.” The average of items was generated, with higher scores representing higher levels of Preparedness. Preparedness demonstrated high levels of internal consistency (*ɑ* = 0.78).

#### Positive and negative affect schedule

Negative Affect was assessed using an adapted form of the Negative Affect subscale of the Positive and Negative Affect Schedule (PANAS) (18), consisting of 20 items, 10 items measuring Negative Affect (e.g., sadness) and 10 items measuring Positive Affect (e.g., hopeful). Participants were asked to indicate the extent to which they had felt an emotion (e.g., sadness) over the past 2 weeks. Participants rated the items from 1 to 100, with 1 being “Never” and 100 being “Always.” For this study, we assessed only the Negative Affect subscale of the PANAS to replicate previous literature which mostly evaluates the relationship between Technology Use and Negative Affect [rather than Positive Affect; see (1)]. This aligns with popular opinion that Technology Use is associated more with negative outcomes than positive outcomes. Negative Affect was scored as the mean across corresponding items. Higher scores indicate higher levels of negative affect. Negative Affect demonstrated high levels of internal consistency (*ɑ* = 0.87).

### Data analysis plan

To test our study hypotheses, we first assessed the moderating influence of Preparedness on the association between the overall Frequency of Technology Use and Negative Affect. To do so, we ran a moderated regression analysis (Model 1) using model 1 of PROCESS (19) within the SPSS Version 27 statistics software. In this model, Frequency of Technology Use, Preparedness, and their interaction term were regressed onto Negative Affect. We then repeated this model, instead looking at the moderating effect of Preparedness on the associations between various forms of technology use and Negative Affect (Models 2–8). One model was run for each form of technology use.

## Results

Table 1 shows the intercorrelations among all study variables, their means, and standard deviations. Normality and linearity assumptions were met and skewness (−1.13 to 1.39) and kurtosis (−1.41 to 1.13) were within the acceptable ranges. It is noteworthy that the correlation between Frequency of Technology Use and Negative Affect is significant but modest. Furthermore, there is notable variation in the strength of association between most indices of technology use and negative affect. For example, the association between Playing Video Games and Negative Affect is not significant (*r* = <0.01, *p* = 0.91), while the association between the two technology use variables that focus on looking at or creating content for social media and Negative Affect are moderate (*r* = 0.21, 0.23, respectively, *p*s < 0.001), and the associations between most other forms of technology and Negative Affect are small (*r* = 0.03–0.11).

**Table 1:** Intercorrelations, means, and standard deviations for all study variables.

	Frequency of technology use	Prepared-ness	Negative affect	Look at social media	Create social media	Browse internet	Play video games	Create digital content	Academics	Communicate with others
Frequency of technology use	–									
Preparedness	0.09	–								
Negative affect	0.21**	−0.19*	–							
Look at social media	0.67**	−0.02	0.21**	–						
Create social media	0.66**	0.02	0.23**	0.55**	–					
Browse internet	0.56**	0.03*	0.10**	0.27**	0.24**	–				
Play video games	0.41**	0.06**	0.00	0.09**	0.04**	0.19**	–			
Create digital content	0.47**	0.06**	0.08**	0.11**	0.30**	0.15**	0.17**	–		
Academics	0.39**	0.11**	0.03*	0.02	0.04**	0.06**	−0.02	0.07**	–	
Communicate with others	0.59**	0.06**	0.10**	0.41**	0.32**	0.28**	0.11**	0.09**	0.07**	–
Mean	2.64	2.86	39.1	2.92	2.09	3.01	3.07	1.54	2.50	3.34
Standard deviation	0.49	0.61	22.86	1.03	0.92	0.82	0.91	0.79	1.13	0.83

*p < 0.05, ** *p* < 0.01.

### Model 1: overall frequency of technology use

**Figure 1:**
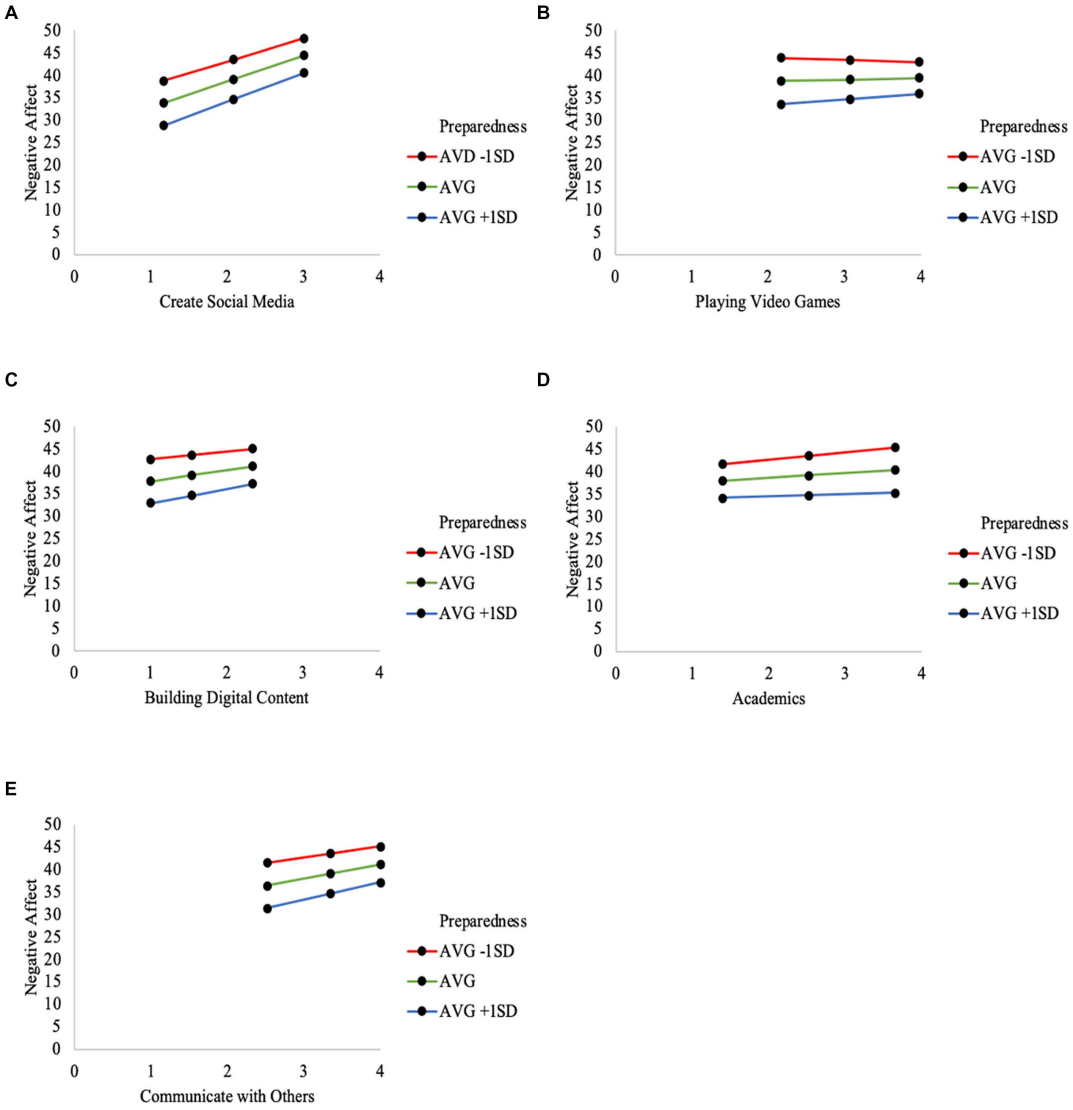
Visualization of the moderated regression results testing preparedness on the association between various types of technology use (**(A)** Create Social Media; **(B)** Playing Video Games; **(C)** Building Digital Content; **(D)** Academics; **(E)** Communicate with Others) and negative affect.

For full model results (see Table 2). Overall Frequency of Technology Use, Preparedness and the interaction term between Frequency of Technology Use and Preparedness were significantly associated with Negative Affect. Figure 1 provides a visualization of the moderating effect.

**Table 2:** Moderated regression results testing the influence of preparedness on the association between technology use and negative affect.

Independent variables	Negative affect
Model 1	
Frequency of technology use	5.48*
Preparedness	−12.27***
Interaction term: frequency of technology use * preparedness	1.72*
*R* ^2^	0.08
Model 2	
Look at social media	2.72*
Preparedness	−8.78***
Interaction term: look at social media * preparedness	0.64
*R* ^2^	0.08
Model 3	
Create social media	2.87*
Preparedness	−9.44***
Interaction term: create social media * preparedness	1.02*
*R^2^*	0.09
Model 4	
Browse internet	3.44*
Preparedness	−6.76***
Interaction term: browse internet * preparedness	−0.13
*R^2^*	0.05
Model 5	
Play video games	−3.98**
Preparedness	−11.86***
Interaction term: play video games * preparedness	1.52**
*R* ^2^	0.04
Model 6	
Build digital content	−0.89
Preparedness	−9.20***
Interaction term: build digital content * Preparedness	1.18*
*R* ^2^	0.04
Model 7	
Academics	3.81**
Preparedness	−4.88***
Interaction term: academics * preparedness	−0.96*
*R* ^2^	0.04
Model 8	
Communicate with others	−0.25
Preparedness	−11.39***
Interaction term: communicate with others * preparedness	1.21*
*R* ^2^	0.05

***p < 0.001, **p < 0.01, *p < 0.05.

### Models 2–8: types of technology use

For full results of Models 2–8 (see Table 2). The frequency of specific technology uses maintained significant associations with Negative Affect for all models, with the exception of Building Digital Content (Model 6) and Communicating with Others Online (Model 8). Preparedness was significantly associated with Negative Affect in all models. Finally, the interaction term generated between specific types of technology use and Preparedness was significant for Creating Social Media, Video Games, Building Tech, technology for Academics, and Communicating with Others Online, but not Looking at Social Media or Browsing the Internet. See Figure 1 for visualizations of the significant interactions.

## Discussion

The current study was designed to deepen our understanding of the relationship between technology use, negative affect, and potential mitigating factors of this relationship. Several findings warrant elaboration. Our general finding that frequency of technology use was associated with negative affect supports previous findings in the literature [i.e., (1)]. However, this finding does not account for nuance that may be reached when considering how prepared the technology users are, or what kind of technology use they are engaged in.

To that end, preparedness showed a protective effect on the relationship between frequency of technology use and negative affect, such that participants with a higher level of preparedness (one standard deviation above the average) yielded lower levels of negative affect at all levels of technology use compared to their peers with lower levels of preparedness (average and one standard deviation below the average). This indicates that, despite associations between higher frequencies of technology use and negative affect, the way technology is used and how prepared students are by their schools may be able to mitigate related negative affect.

At all levels of technology use, students with high levels of preparedness reported lower levels of negative affect than their peers with low levels of preparedness. It is noteworthy that when youth with high levels of preparedness are using very high levels of technology, they report similar levels of negative affect as youth with low levels of preparedness when they are using low levels of technology use. Prior neurobiological work has shown that adolescence is a developmental period accompanied by increased risk-taking (20), and it can be safely assumed that this habit of risk-taking would persist in a digital setting. Supported by neurobiological literature, the practical application for this finding is clear: students that feel adequately prepared by their school to use technology can use technology at moderate and even high frequencies with less risk of experiencing the levels of negative affect that their less prepared peers face. This is supported in previous literature (11), and seems especially important as cases of remote learning and remote work using technology become increasingly popular over time. Proper intervention, namely preparedness, can potentially mitigate adverse effects caused by heightened risk-taking. Preparing students to succeed in digital environments, whether it be while they are in school, on social media, or for future entry into the workforce is crucial to protecting against negative affect.

When analyzing the different types of technology use and their relationship with negative affect, we found that 5 of the 7 types had their relationship with negative affect change when preparedness was considered: 1. Creating Social Media, 2. Playing Video Games, 3. Building Digital Content, 4. Academics, and 5. Communicating with Others. Noticeably, these types of technology use suggest some form of active engagement. The two types of technology whose relationships with negative affect were not significantly shifted by preparedness were: 1. Browse the Internet and 2. Looking at Social Media, which notably are more passive forms of engagement. Although there is little to draw from the literature to explain these findings, we speculate that preparedness moderated the types of technology that have clear, teachable skills that students can learn to engage in more safely. Conversely, schools may not yet be at a point where they can adequately prepare students to browse the internet or look at social media passively in safe, adaptive ways. This would call for more efforts in preparing students to use these forms of technology in moderate amounts, and would suggest that some level of active usage is necessary when using technology to prevent increases in negative affect. While more research is needed to replicate these findings, this provides some evidence that preparing students not just to consume technology, but to create and engage with it, may be one avenue for diminishing consequent negative affect. This finding is novel and further research should attempt to replicate the various effects preparedness has on active and passive technology use more directly than we did in the current study.

The findings in this study are novel and have important implications for the application of technology-based learning in schools, as well as technology use in general, but it is not without its limitations. One limitation is that we asked participants to self-report on their technology use and negative affect, a method that, although popular in the literature, can be unreliable and prone to participant biases (21). In future work, a better means of quantifying technology usage may be to ask participants to examine their device-usage logs, such as Apple Screen Time or an alternative experimental approach, in conjunction with our self-report measure. It may also be helpful to capture how, specifically, adolescents spend their time using different types of technology, as opposed to only frequency of time spent. We also note that the R2 values of our significant models, and therefore the level of variance in negative affect that we account for, is small. We highlight that accounting for between 4 and 9% of the variance in an individual’s level of negative affect during adolescence is a meaningful contribution to the field, however, we also encourage further study of these phenomena in order to replicate and contextualize these findings. Another limitation is that these data were collected at one point in time, and may not be representative of participants’ long term technology usage or negative affect. These results are also correlational, and therefore we cannot determine causation from this specific study. Future studies might distribute these measures at multiple points over time to replicate these findings in a way where causality may be determined. We can also note that the sample of schools who were part of this study all had the internet access and technological resources for students to complete surveys online which may skew the frequency of technology use compared to general averages. Additionally, this study focused primarily on the relationship between technology use and negative affect, with little mention of positive affect. Future reports should address adolescent technology use and positive affect as its own research topic, as prior literature suggests negative and positive affect represent nearly discrete dimensions (22, 23) which would possibly interact with different forms of technology use in unique ways. Finally, examining whether school level characteristics influenced the associations in this study were beyond the scope of this paper. We encourage future studies that are able to provide multi-level examinations of how these dynamics unfold across important school-level factors, such as school size, urbanicity, and average income level.

With increasing interest in the relationship between technology use and adolescent affective experiences, and mixed findings in the literature, we sought to elucidate the association between the two constructs. Our findings suggest a moderate relationship between time spent using technology and negative affect, cutting across types of technology engagement. These findings also highlight the importance of investing time in schools to prepare students to succeed in using technology, as well as which types of technology most benefit from this preparation. In addition to the time investment at the school level, these findings can aid educational policymakers in their decisions to adequately allocate funds that support schools in providing students with high levels of technological preparedness. We hope that our findings will encourage a higher emphasis on adolescent affective well-being when using technology and evolve the conversation from overall “screen time” to more nuanced discussions about various types of technology use.

## Data availability statement

The raw data supporting the conclusions of this article will be made available by the authors, without undue reservation.

## Ethics statement

The studies involving human participants were reviewed and approved by the Yale University Human Subjects Committee. Written informed consent from the participants’ legal guardian/next of kin was not required to participate in this study in accordance with the national legislation and the institutional requirements.

## Author contributions

JH contributed to the conception and design of the original study. SM, TT, KD, and JH contributed to the data collection, data cleaning, interpretation of findings, and writing of the manuscript. SM and KD contributed to data analyses. All authors contributed to manuscript revisions and read and approved the submitted version.

## Funding

This work was a part of a research study entitled The inspirED Process: Empowering Youth to Launch Peer Outreach Projects that Promote Healthy Social Media Habits Grant (PI: JH) funded by the Susan Crown Exchange.

## Conflict of interest

The authors declare that the research was conducted in the absence of any commercial or financial relationships that could be construed as a potential conflict of interest.

## Publisher’s note

All claims expressed in this article are solely those of the authors and do not necessarily represent those of their affiliated organizations, or those of the publisher, the editors and the reviewers. Any product that may be evaluated in this article, or claim that may be made by its manufacturer, is not guaranteed or endorsed by the publisher.
